# Current use of measurement instruments by physiotherapists working in Germany: a cross-sectional online survey

**DOI:** 10.1186/s12913-018-3563-2

**Published:** 2018-10-23

**Authors:** Tobias Braun, Alina Rieckmann, Franziska Weber, Christian Grüneberg

**Affiliations:** Hochschule für Gesundheit (University of Applied Sciences), Department of Applied Health Sciences, Division of Physiotherapy, Gesundheitscampus 6-8, 44801 Bochum, Germany

**Keywords:** Measurement instrument, Outcome measurement, Physical therapy, Physiotherapy, Rehabilitation, Evidence-based practice, Cross-sectional survey

## Abstract

**Background:**

The use of measurement instruments in physiotherapy has been recommended in clinical practice guidelines to improve evidence-based practice. The aims of the study were (a) to describe the current use of measurement instruments by physiotherapists working in Germany and (b) to investigate the facilitators and barriers to use measurement instruments.

**Methods:**

This cross-sectional study used a nationwide online survey, which was accessible to all physiotherapists working in Germany.

**Results:**

In total, 522 adult physiotherapists working in Germany completed the questionnaire. The mean age of the respondents was 38 years, 63% were female, and 53% had >10 years of work experience.

Thirty-one percent of the respondents used measurement instruments in ≥80% of their patients, and 26% used measurement instruments in ≤20%. Measurement instruments were used for diagnostic and prognostic purposes by 69% and 22% of respondents, respectively. The three most frequently reported measurement instruments were “goniometer” (*n* = 254), some kind of a “visual/numeric analogue scale” (*n* = 139), and the “manual examination of muscle-strength” (*n* = 54). Seven of the 13 most stated measurement instruments measure activities or participation.

The most important facilitator was physiotherapists’ positive attitudes towards measurement instruments. Two out of three respondents reported having sufficient knowledge and skills to apply measurement instruments in clinical practice. The most pronounced barriers were insufficient additional financial compensations and requiring extra time to document test scores. Seventy-eight percent of the respondents could imagine using an electronic device for a user-friendly patient health record system in clinical practice.

**Conclusions:**

The limited use of measurement instruments reported by physiotherapists working in Germany appears to be due to organisational issues, in combination with a lack of knowledge and skills needed to apply the measurement instruments, rather than due to individual or managerial reasons. To support the use of measurement instruments, sufficient time resources and adequate financial compensation are required. Educational approaches should focus on imparting patient-centred and patient-reported outcomes to quantify activities and participation. Electronic patient health record systems have potential to facilitate the application of standardised measurement instruments if the barriers identified in this survey are addressed properly.

**Electronic supplementary material:**

The online version of this article (10.1186/s12913-018-3563-2) contains supplementary material, which is available to authorized users.

## Background

Measurement instruments (MI) are tools for measuring various aspects of a person’s health status, such as impairments, activity limitations, participation, and quality of life [1]. MI can be used for diagnostic purposes, for measuring the outcome of health care interventions, and for determining prognoses. Thus, the use of MI is an inherent part of evidence-based practice, and MI are considered to be tools that support the clinical decision-making process [2–5]. The use of MI, either self-reported or performance-based, has been recommended for rehabilitation professionals in many clinical practice guidelines [6–8]. In the literature, the term “outcome measure” is used frequently for a MI that is used to determine the change in ability from before to after an intervention [9, 10]. Jette et al. reported that “measures, in general, are standardised in that they use closed-ended questionnaire formats or specific protocols for implementation, provide scores that allow quantitative assessment of ability, and have been evaluated for their psychometric properties” [10].

However, cumulative evidence from various studies conducted in different countries, such as New Zealand [11], Canada [12, 13], The Netherlands [14, 15], Switzerland [16], Austria [17], Saudi Arabia [18], the U.S. [10, 19], Ireland [20] and Australia [21], indicate limited use of MI by physiotherapists [9]. The most relevant barriers reported in the scientific literature are physiotherapists’ level of knowledge and competence in the use of MI; problems related to changing behaviour; structural restrictions, such as a lack of time; and the unavailability and limited feasibility of MI [9]. Therefore, there is an urgent need for effective strategies to implement and facilitate the use of MI in physiotherapy, especially since there is evidence that most therapists have a positive attitude towards MI and are convinced of their advantages in clinical care [2, 9].

Some approaches to facilitate the use of MI in physiotherapy have been proposed and examined. For example, van Peppen et al. (2009) [22] and Gutierrez Panchana et al. (2018) [23] reported positive effects of tutor-guided educational sessions on the actual use of instruments by physiotherapists who were involved in stroke management. Another important approach is the development of core outcome sets, which can be used for certain groups of patients [24]. The implementation of electronic patient health record systems is a new and promising approach to reinforce the use and communication of MI [25].

It has been reported that physiotherapists with an university-based professional degree are more likely to use MI than therapists with a non-academic or lower education level [9, 11, 18]. In Germany, an academic level of physiotherapy education is not required and most physiotherapists graduate from a vocational school (polytechnic level; so called “Berufsfachschulen”) [26]. However, the number of physiotherapists in Germany with a higher education increases with 3% of approximately 192.000 physiotherapists having a Bachelor’s or a Master’s degree in 2018 [26, 27].

There is some evidence on factors that influence the use of MI in clinical practice. Barriers to use MI were time restrictions, a lack of funding or excessive costs of outcome measures [9]. In contrast to many other European countries, in Germany physiotherapists do not receive any additional reimbursement or paid time for the examination and assessment of the patient.

Within this professional structure and in light of recent developments, it is very useful to have valid information on the use of MI in physiotherapy practice in Germany, because determining specific facilitators and barriers to use MI will assist in developing interventions to enhance uptake of MI by physiotherapist. We further assume that facilitators and barriers within the German context of practice are likely to differ from previous research. The objective of this study was to describe the current use of MI in physiotherapy practice in Germany, in combination with the facilitators and barriers for application.

## Methods

### Design

In this cross-sectional study, an online survey was used to analyse the current use of MI in physiotherapy in Germany, as well as facilitators and barriers of application. All respondents (physiotherapists) participated anonymously and voluntarily. By completing the survey, participants gave informed consent for data analysis and publication. The study was performed according to the ethical principles described in the Declaration of Helsinki. Reporting followed the Strengthening the Reporting of Observational Studies in Epidemiology (STROBE) guideline for observational studies [28] and the Checklist for Reporting Results of Internet E-Surveys (CHERRIES) [29].

### Measurement instrument

We used a pre-existing questionnaire, which had been designed to assess the use of MI and the facilitators and barriers to the utilisation and implementation of MI. The process of questionnaire compilation has been described in detail elsewhere [30]. Briefly, a review of the existing international literature on the usage of MI in clinical care was conducted [10, 11, 14, 15, 20, 21, 31–34]. Relevant facilitators and barriers for implementation were identified and ordered into six categories: (1) the attitudes and beliefs of the therapist, (2) skills and knowledge, (3) therapeutic setting, (4) organisational structures, (5) clinical reasoning process and (6) interprofessional approach.

The questionnaire had been used in a previous study, including physiotherapists working with neurological patients in Austria [30]. For the present study, the questionnaire was reviewed, revised and extended to improve readability and face validity. The final questionnaire consisted of four sections (described below) and included more than 50 items (questions and statements). The complete German-language questionnaire is given in Additional file 1.

### First section

Physiotherapists were asked to provide personal and work-related information, such as their country and federal state of employment, age, gender, degree of education, further education and training, recent work setting, quantity and quality of patients treated per week, and work experience.

### Second section

This section dealt with the usage of MI in clinical practice. Initially, the definitions of “assessment” [35] and “interdisciplinary” [36] were given (see Additional file 1 for details). Following this, physiotherapists were asked to select the purposes of MI usage from a given list, including diagnostic and prognostic purposes. Other items assessed the number of instruments used in daily practice and the rate of patients assessed with MI (0% to 100%).

We used the German word “Assessment” in the questionnaire, which is an englishism/translation used for “measurement instrument” in the German-speaking physiotherapy community. The term “Assessment” is very common in Germany because of a popular German textbook-series on MI in rehabilitation, in which the term “Assessments” is used by the authors consistently [35, 37–39].

Respondents were asked to list up to six MI (“Assessments”) that were obligated by their employer and the six instruments applied most frequently in their clinical care. The employer could be a hospital’s administration, the head of a physiotherapy department, or the head of an outpatient clinic. Some participants listed more than six instruments by writing > 1 instrument’s name in a response-field. For analysis, the number of instruments per respondent was limited to six instruments (according to the order of listing). During the process of data analyses, we recognized that some participants did not only report “measurement instruments”, but also devices (e.g. measuring tape), outcomes (e.g. range of motion) or unclear statements such as “questionnaire”. Thus, a statement/“instrument” was categorised as a “measurement instrument” if it was described in one of the German-language textbooks on MI published by Schädler et al. [37], Oesch et al. [38] and Büsching et al. [39]. Most MI described in these books can be considered as “standardized” according to the definition by Jette et al. [10] because of specific protocols for implementation, scores that allow quantitative assessment of ability, and an evaluation of psychometric properties.

### Third section

This section was set up to assess information on facilitators and barriers for the usage of MI in physiotherapy. Participants expressed their opinions and attitudes on several statements on a 5-point Likert scale: “agree”, “rather agree”, “neutral”, “rather disagree” and “disagree”. The response option “I cannot judge” was provided for each statement. Statements were separated into the six categories described above. Additionally, a seventh category was used to gain information on the facilitators and barriers for using an electronic patient health record system in clinical care. This was done by means of an open-ended question.

### Fourth section

This part dealt with potential training needs in the clinical application of MI and was added to the existing questionnaire [30]. Results will be reported elsewhere (in preparation).

To generate the online version of the questionnaire, the software “EvaSys SurveyGrid” was used (Electric Paper Evaluationssysteme GmbH, 21,337 Lüneburg, Germany; https://surveygrid.evasys.de/start). The survey was accessible online only via a “survey homepage” that was launched to provide access to the questionnaire. The whole survey was distributed on seven separate pages/screens. Participants were able to review and change their answers.

Physiotherapists could complete the survey without any restrictions, such as a password or registration. For all questions and statements, participants were forced to make a choice to either get to the next level or to complete the whole questionnaire. An additional response field for free text was provided for many items in the questionnaire. All the survey’s items were offered in a standardised, unaltered order.

### Participants

The voluntary online survey was accessible for all German-speaking physiotherapists (unrestricted public internet link and sample of convenience). In the present study, we only included questionnaires completed by adult (≥18 years), graduated physiotherapists working in clinical care in Germany. We excluded questionnaires from respondents who were not working in Germany, those who reported to be undergraduates or trainees, those who reported not to work with patients at the time of survey conduction, and those who reported not to have any diploma in physiotherapy, such as sport therapists.

### Procedures and data collection

The online questionnaire was launched for 14 weeks, starting on November 13th, 2014 and ending on February 20th, 2015. The survey homepage provided a short description of the aims, the estimated conduction time, the launch period, the research team (investigators) and some short instructions on how to complete the survey.

We used diverse media and communication channels to inform German-speaking physiotherapists about the survey. For this purpose, a short “advertisement” was created. This advertisement included a description of the aims, procedures and contact information of the persons responsible of the project. In addition, the advertisement included a link and a Quick Response (QR) code leading to the survey homepage. A picture of the research team was provided optionally. This advertisement was published via different media by the institutions, professional societies and journals listed in Additional file 2.

A survey invitation e-mail was sent to all physiotherapy departments, outpatient clinics and institutions that were listed as cooperation partners of our university (*n* = 57). Usually, this includes the head of the institution/department and/or the physiotherapist responsible for the practical training of students. We asked to distribute the survey link and the project to as many colleagues as possible (“snowball principle”). A reminder was send to all cooperation partners 8 weeks after the initial invitation. We did not define a minimum sample size, but aimed to include as many physiotherapists throughout all regions of Germany as possible. No incentives were offered for participation.

### Statistical analysis

Data from the online survey were saved as an Excel file by SurveyGrid. The data were transferred to and analysed with SPSS Version 21.0 statistical software (SPSS Inc., Chicago, IL, USA). No weighting of items or propensity scores were used. Summary descriptive statistics (i.e. mean values with 95% confidence intervals) were computed for the socio-demographic data of the respondents and the MI used.

The results on barriers and facilitators of MI usage in clinical practice are presented as frequencies of responses. In addition, we described these results in a narrative way, for which the two response categories “agree” and “rather agree” (agreement) and the two response categories “rather disagree” and “disagree” (disagreement) were collapsed.

The impact of gender, educational level, work experience, work setting, extent of work, number of patients treated per week, and main type of patients treated on the use of MI in clinical care was estimated using logistic regression analyses with generalised linear models. Independent variables with a *p*-value below 0.10 in univariate logistic regression analysis were included in the multivariate logistic regression model to estimate the adjusted impact of MI application (dependent variable). *P*-values < 0.05 were considered significant. Goodness-of-fit was assessed with a Hosmer-Lemeshow test. All analyses were exploratory, thus *p*-values may be inflated and odds ratios need to be interpreted with caution.

To define the dependent variable (frequent or infrequent use of MI), we pooled all participants who reported to use MI in 0%, 10% or 20% of patients (infrequent use, *n* = 137) and all participants who reported application in 80%, 90% or 100% of patients (frequent use, *n* = 163). Participants who reported to use MI in 30% to 70% of patients were excluded from these analyses (*n* = 222). The selection of independent variables was informed by the international literature [9–11, 18]. For the variable “work setting”, participants were dichotomised to “inpatient” (working predominantly in a hospital or a rehabilitation clinic, *n* = 97) and “outpatient” (participants working predominantly in an outpatient clinic/private practice, *n* = 397). Participants who worked in both of these settings without indication of preponderance, and participants who worked predominantly in another setting, were classified as “no primary setting of work/other” and excluded from these analyses (*n* = 28). For the variable “main type of patients”, participants were asked to indicate the relative amount of patients they have treated according to the four disorder types “musculoskeletal”, “neurological”, “internal medicine” and “mixed disorders” on a 0–100% scale for each category. The four given values should sum up to 100%. Participants who reported to treat at least 50% of patients of one category were classified as “specialised” according to each category (musculoskeletal or neurological/internal medicine).

The five most frequently reported MI are presented for the whole sample and according to the subgroups “work setting” and “main type of patients”.

We analysed the open-ended question on the facilitators and barriers to use an electronic patient health record system in clinical care by using a framework analysis [40]. This approach enabled the investigation of a priori objectives while also allowing new themes to emerge from the data. One researcher (AR) transcribed and coded each transcript and another (FW) undertook the initial coding of a selection of transcripts. Similarities and differences between the coding labels and definitions were discussed, and the coding framework was refined and applied to the answers. The coding process was iterative; emergent codes were added to the framework and contributed to the development of themes across the answers by the respondents. Codes were synthesized and grouped according to the dominant emergent themes. These themes were mapped onto a framework developed by Wensing et al. [40], which specified six levels of factors that facilitate or impede implementation success: the innovation, the individual professional, the patient, the social context, the organisational context, and the economic and political environment.

## Results

In total, 595 surveys were completed by adult physiotherapists. The present study reports on the data from 522 (87.7%) surveys completed by respondents who reported to work with patients in clinical care in Germany. Surveys completed by respondents from Switzerland (*n* = 47; 7.9%), Austria (*n* = 8; 1.3%) or other countries (*n* = 2; 0.3%) were excluded. Details of the determinants of respondents are given in Table 1. There was no missing data.

**Table 1:** Determinants of responding physiotherapists (*n* = 522)

Determinant	Value
Sex [male/female]	194/328 (37%/63%)
Age in years, mean	38.2 ± 11.5 (19–67)
18–30	188 (36%)
31–40	111 (21%)
41–50	121 (23%)
50+	102 (20%)
Highest degree of education	
Diploma (vocational school)	368 (70%)
Bachelor/diploma (university)	110 (21%)
Master	38 (7%)
Higher academic degree	6 (1%)
Further education/training, median^a^	2 (1–3)
None	45 (9%)
Medical exercise training	293 (56%)
Manual therapy	333 (64%)
Manual lymphatic drainage	314 (60%)
Vojta therapy	19 (4%)
Proprioceptive neuromuscular facilitation (PNF)	120 (23%)
Bobath/Neurodevelopmental Treatment (NDT)	130(25%)
At least one other training	149 (29%)
Work experience in years	
< 1	48 (9%)
1–3	63 (12%)
4–10	132 (25%)
11–15	56 (10%)
> 15	223 (43%)
Primary setting of work	
Hospital or rehabilitation clinic (inpatient)	97 (19%)
Outpatient clinic/(private) practice	397 (76%)
No primary setting of work or “other”	28 (5%)
Number of working hours per week, median	38 (30–40)
1–30	172 (33%)
31–40	240 (46%)
41+	110 (21%)
Number of patients per week	
1–5	46 (9%)
6–10	89 (17%)
11–15	132 (25%)
16–20	172 (33%)
21–25	52 (10%)
> 25	31 (6%)
Main type of treated patients	
Musculoskeletal	381 (73%)
Neurological or internal medicine	53 (10%)
Mixed	88 (17%)
Age group of patients^a^	
Young children < 6 years	63 (12%)
Children 6–13 years	125 (24%)
Adolescents 14–17 years	220 (42%)
Adults 18–65 years	489 (94%)
Older adults > 65 years	386 (74%)

Values are the total numbers (percent) or indicated otherwise. Mean values are given with the standard deviation (range), and median values are given with the interquartile range. ^a^ Multiple answers possible

The mean age of participants was 38 years, and approximately two out of three had a non-academic diploma in physiotherapy. Most respondents were employed in an outpatient practice (76%), treated > 10 patients per week (74%) and had a high level of work experience (53% had > 10 years). The majority of respondents reported to work with adult patients and with patients referred to physiotherapy due to musculoskeletal disorders (73%). The figure in Additional file 3 illustrates the distribution of the respondents across the 16 German federal states.

### Usage of measurement instruments

**Fig. 1:**
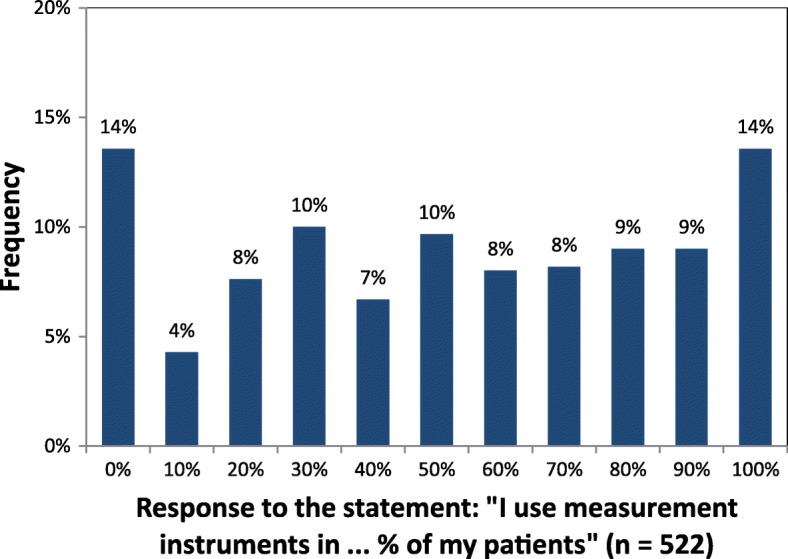
Usage frequency of measurement instruments

The use of MI in general was indicated by 86% of respondents (449/522). Figure 1 illustrates the usage frequency of MI. In total, 163 (31%) respondents used MI in ≥80% of their patients, and 137 (26%) used MI in ≤20% of patients.

MI were used for diagnostic and prognostic purposes by 361 (69%) and 113 (22%) respondents, respectively. With respect to the purpose of instrument usage along the rehabilitation course, the reported frequencies were: initial/admission assessment (383; 73%), intermediate/re-evaluation (334; 64%), and final/discharge assessment (350; 67%). Thirty-three respondents reported at least one other purpose for instrument usage, including better documentation (*n* = 6), inter-professional communication (*n* = 6), within the clinical reasoning process (*n* = 5), as a pre-post-test within one session (*n* = 4), research (*n* = 4), to define treatment goals and strategies (*n* = 3), to increase patient motivation (*n* = 3), for communication with patients (*n* = 2), and for deduction (*n* = 1).

Respondents were asked to list the six most frequently applied MI. In total, 1497 statements were given, including 267 different MI, methods and devices.

The 21 most frequent statements, each with ≥10 reports, are listed in the table in Additional file 4. Of those, 62% (*n* = 13) are MI and eight statements were categorised as devices, outcomes, methods or “unknown”, such as “measuring tape”, “range of motion” or “questionnaire”.

Of the 13 MI, 46% (*n* = 6) are solely measures of body functions and structures, 38% (*n* = 5) are solely measures of activities and participation, and 15% (*n* = 2) of the measures assess body functions, body structures and activities (finger-floor distance and Disabilities of the Arm, Shoulder and Hand Score; DASH/quick-DASH). The three most frequently reported MI were “goniometer” (49%), “visual analogue scale”, “numeric analogue scale” or “numeric rating scale” (27%), and the manual examination of muscle-strength (10%).

One hundred and fifty-one (29%) respondents reported that the application of MI was made compulsory by their employer. We asked these participants to list up to six MI that were predefined. A total of 587 statements were given, including 166 different MI, methods and devices (table in Additional file 5: The 21 most frequent statements with ≥7 reports each). The three most frequently reported MI were “goniometer” (15%), “visual analogue scale”, “numeric analogue scale” or “numeric rating scale” (9%), and the Timed Up and Go test (6%).

**Fig. 2:**
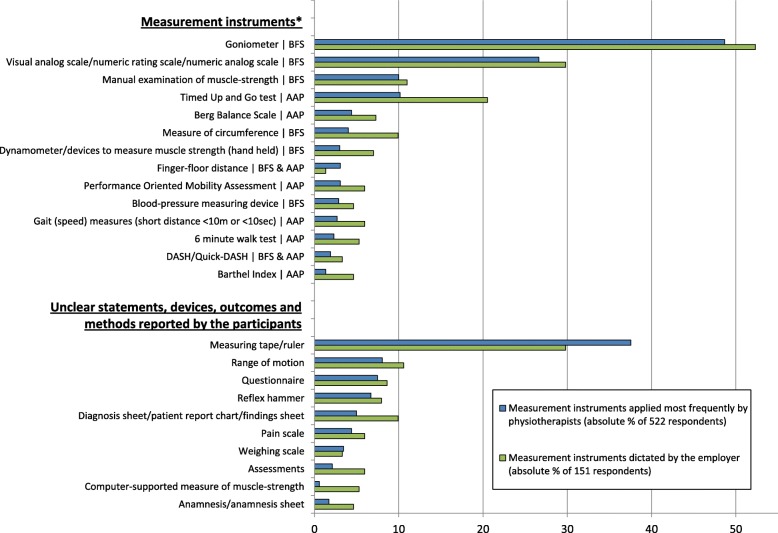
Frequency of statements concerning the measurement instruments (or methods/devices) that were applied most frequently by respondents and dictated most frequently by the employer. Abbreviations: BFS = body functions and structures; AAP = activities and participation; DASH = Disabilities of the Arm, Shoulder and Hand. (* described in one of the German-language textbooks on measurement instruments [37–39])

Figure 2 illustrates the most frequently reported MI and the most frequently reported devices, methods and unclear statements reported by the respondents, together with the most frequent instruments dictated by the employer.

**Fig. 3:**
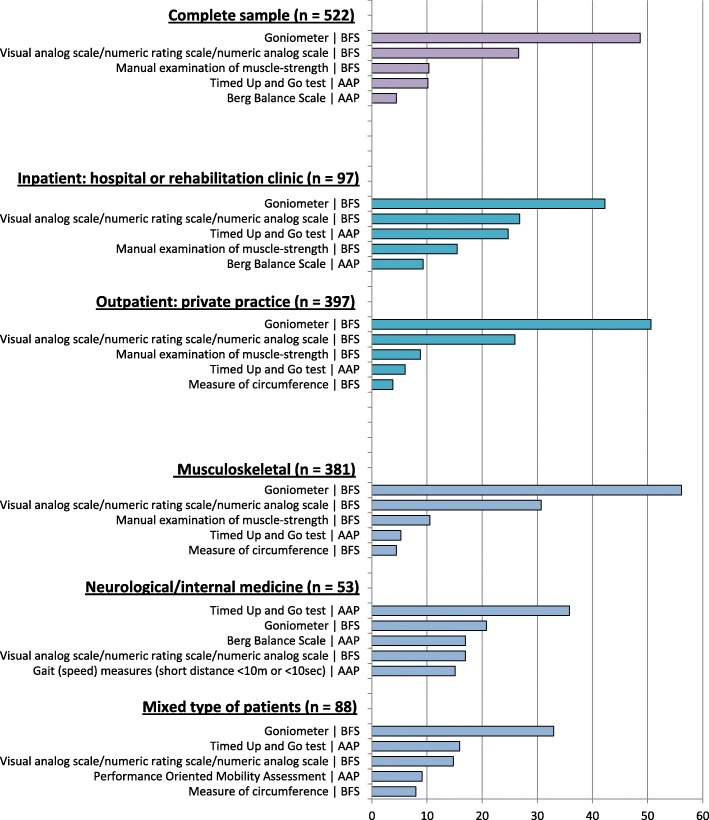
Frequency of the 5 most frequently used measurement instruments, described in one of the German-language textbooks on measurement instruments [37–39], by the complete sample of participants and according to the subgroups “work setting” and “main type of patients”

Figure 3 illustrates the five most frequently used MI by the complete sample of participants and according to the subgroups “work setting” and “main type of patients”.

### Facilitators and barriers

All absolute and relative response frequencies on the facilitators and barriers are given in the table in Additional file 6.

### Attitudes and beliefs of the therapists

**Fig. 4:**
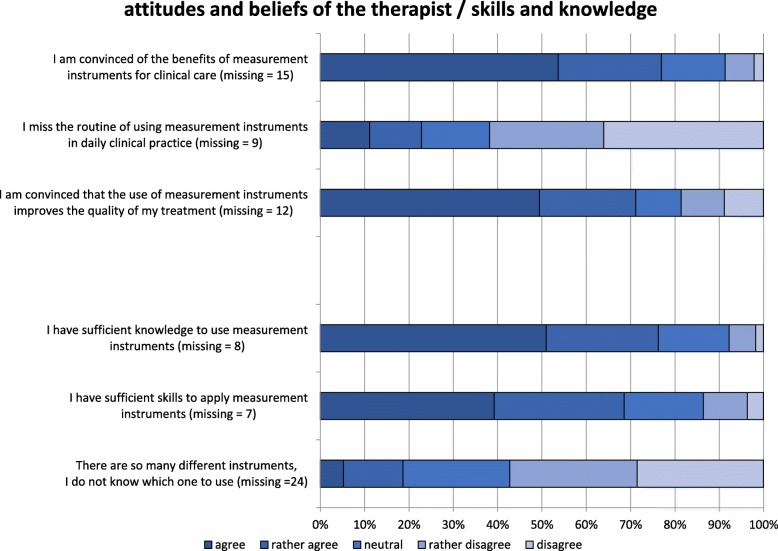
Facilitators and barriers to the use of measurement instruments in physiotherapy in the categories of “perspective of the therapist” and “skills and knowledge” (*n* = 522). Missing values (m) represent the “cannot assess” option

Respondents generally reported a positive attitude towards MI (Fig. 4). Seventy-five percent of respondents were convinced that MI have clinical benefits and improve the quality of physiotherapy treatments. In 22% of respondents, we identified a lack of routine in using MI in daily clinical practice.

### Skills and knowledge

Most respondents reported having sufficient knowledge (75%) and skills (68%) to apply MI (Fig. 4). The high number of available instruments seems not to be a barrier for the selection of MI in clinical care.

### Therapeutic setting

**Fig. 5:**
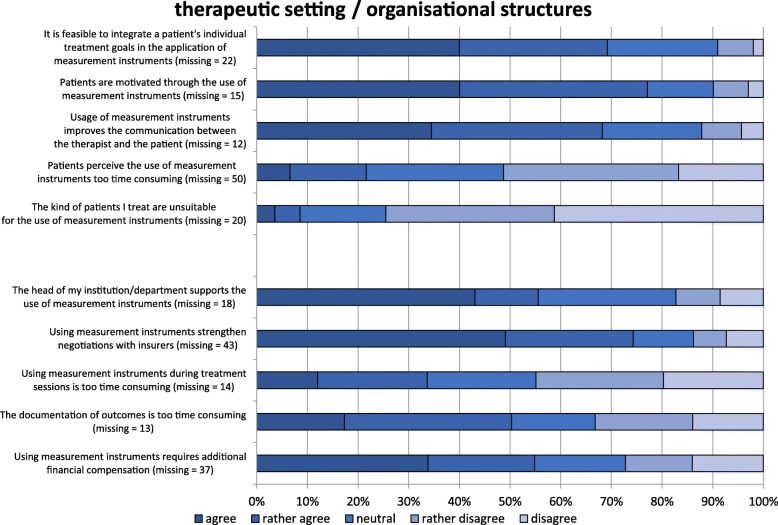
Facilitators and barriers to the use of measurement instruments in physiotherapy in the categories of “therapeutic setting” and “organisational structures” (*n* = 522). Missing values (m) represent the “cannot assess” option

The facilitators and barriers in this category are given in Fig. 5. Approximately two out of three therapists (66%) thought that individual patient goals could be well integrated into MI. Of the respondents, 75% and 67% agreed that MI can improve the patients’ motivation and communication between the therapist and the client, respectively. At least 20% of respondents reported that patients generally felt that administration times spent on MI are too time-consuming. In general, most respondents (72%) considered their patients to be suitable for MI application.

### Organisational structures

The results for this category are given in Fig. 5. The head of the department supported the usage of MI for 54% of respondents. It was not supported in almost one out of five institutions. Most respondents reported that MI can serve as a good basis for argumentation towards health care insurances and other sponsors, such as accident insurances.

The application of MI within the treatment time was judged to be too time-consuming by 33% of respondents, whereas 44% of respondents disagreed with that statement. The documentation of clinical outcomes was too time-consuming for 49% of therapists. However, this barrier was not relevant for one third of respondents. Fifty-one percent of respondents thought that using MI required additional financial compensation.

### Clinical reasoning process

**Fig. 6:**
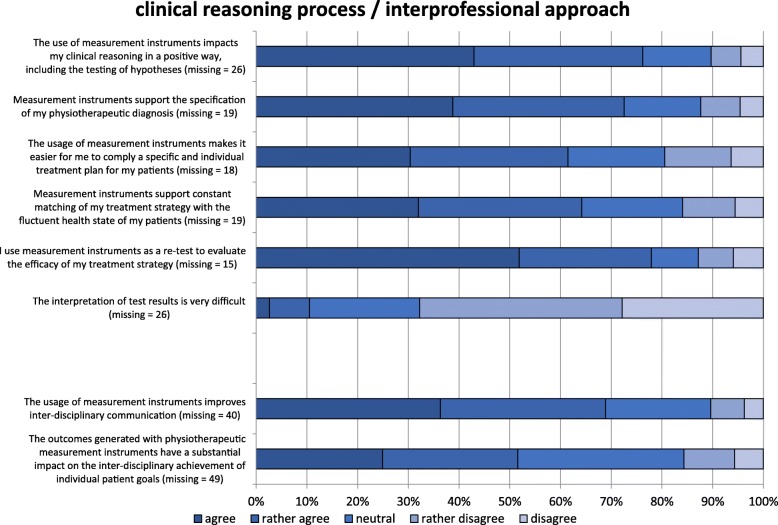
Facilitators and barriers to the use of measurement instruments in physiotherapy in the categories of “clinical reasoning process” and “inter-professional approach” (*n* = 522). Missing values (m) represent the “cannot assess” option

Figure 6 illustrates the results of this category. Seventy-two percent of respondents agreed that MI have a positive influence on the clinical reasoning process. Most of the therapists thought that MI could help in specifying the therapeutic diagnosis (70%), compiling a treatment plan (59%), and adjusting the treatment strategy towards the actual health state of the patient (62%). However, approximately 10% to 19% of respondents did not see any benefits in MI for the clinical reasoning process. The interpretation of the test results seemed to be no barrier within this sample.

### Inter-professional approach

Sixty-four percent of physiotherapists thought that MI could improve inter-professional communication (Fig. 6), 19% were inconclusive on this topic, and 10% disagreed. Approximately half of the respondents (47%) agreed that physiotherapist’s test results have a substantial impact on the achievement of inter-professional treatment goals.

### Additional facilitators and barriers

The respondents mentioned additional facilitators and barriers for implementation of MI in physiotherapy, which will be reported elsewhere (in preparation).

### Participant characteristics influencing the use of measurement instruments

The variables gender (*p* = 0.45), work experience (*p* = 0.57), working hours (*p* = 0.11), and type of patients (*p* = 0.35) were not independently associated with frequent use of MI, whereas the variables educational level (*p* < 0.001), main setting of work (*p* < 0.001), and the number of patients treated per week (*p* = 0.008) were independently associated. In the multivariate model, only the variables educational level and main setting of work remained significant (Table 2). The Odds of a frequent use of MI are listed in Table 2, where each factor is adjusted for the remaining variables in the model. Compared with physiotherapists with a non-academic degree, those with a university-based professional degree were nearly five times more likely to use MI frequently and those working mainly in inpatient settings were approximately four times more likely to use MI in ≥80% of patients. The Chi-square goodness-of-fit test (Hosmer-Lemeshow test: chi^2^ = 2.508; *p* = 0.868) and Nagelkerke R^2^ generalized coefficient of determination (R^2^ = 0.230) indicate that these factors explained 23% of the variance in the regression model. The significant influence of the number of patients treated (univariate analysis: *p* = 0.008) was no longer evident in the multivariate regression analysis.

**Table 2:** Odds of frequent use of measurement instruments by participants and practice characteristics (results of the multivariate logistic regression analysis^a^)

Factor	Use of measurement instruments in ≥80% of patients	Odds Ratio	95% CI		*P*-value
			Lower	Upper	
Professional degree					< 0.001
Non-academic (vocational school)	45% (99/220)	Reference			
Academic (Bachelor, Master or PhD degree)	80% (64/80)	4.81	2.49	9.29	
Main setting of work					< 0.001
Outpatient clinic / private practice	47% (106/226)	Reference			
Inpatient (hospital or rehabilitation clinic)	79% (45/57)	3.96	1.90	8.27	
Number of patients treated per week					
1–5 patients	79% (23/29)	3.48	0.91	13.24	0.07
6–10 patients	56% (30/53)	1.40	0.46	4.21	0.56
11–15 patients	51% (36/71)	1.51	0.52	4.34	0.45
16–20 patients	55% (52/94)	2.05	0.74	5.69	0.17
21–25 patients	40% (12/30)	1.10	0.33	3.69	0.88
26+ patients	43% (10/23)	Reference			

Abbreviations: *CI* confidence interval

^a^Goodness-of-fit statistics: chi^2^ = 2.508; *p* = 0.868; *R*^2^ = 0.230

Note that each factor is adjusted for the remaining variables in the model

### Facilitators and barriers to use an electronic patient health record system

Seventy-eight percent (*n* = 422) of the respondents could imagine applying a user-friendly health record system. In total, 309 participants (57%) made an evaluable statement to the open-ended question about facilitators and barriers: 5% (*n* = 15) mentioned only facilitators, 74% (*n* = 228) mentioned only barriers and 21% (*n* = 66) mentioned both facilitators and barriers. The answers were classified into different subcategories.

For the interpretation of all the statements, a checklist by Wensing et al. [40] was used to document the statements by using established categories. All facilitators and barriers were categorised in one of the following superordinate points: individual level, professional interaction, organisational level, and factors related to structures (figure in Additional file 7). Furthermore, we assigned every statement to a sub-category (table in Additional file 8).

The most commonly mentioned facilitator for using an electronic device with a health record system was the improvement of organisational processes, especially access to patient data and documentation. Furthermore, the improvement and efficiency of therapeutic processes and the aspect of time saving were mentioned. The most frequently mentioned barriers for the implementation of an electronic health record system were costs and time. Further barriers were the need for special training due to the users’ lack of information-technology knowledge and issues with user compliance. Other crucial barriers seen by the respondents were issues with data protection, logistical efforts, the accessibility of data, no remuneration for additional work, hygiene, and constraints in the interaction between patients and therapists.

## Discussion

The primary objective of this study was to describe the current use of MI in physiotherapy in Germany, together with the facilitators and barriers of application. A sample of 522 physiotherapists working in clinical care in Germany participated in this nationwide survey. The key finding is that only 31% of respondents used MI frequently (for ≥80% of their patients), and 26% of respondents reported using MI very rarely (for ≤20% of patients). Furthermore, only 15% of participants reported using MI for every patient, and 14% did not use MI at all. This result is surprising, since the use of MI has been advocated for use by physiotherapists for many years and is recommended in nearly every clinical practice guideline [6–8]. However, 86% of the respondents used MI, and this figure is higher than usage frequencies reported in most former studies. Swinkels et al. (2011) [14] reported the use of standardised measures by 72% (private practice) and 97% (nursing home) of physiotherapists in a Dutch survey. In contrast, figures were lower for New Zealand in 2008 (40%, use of back pain-related outcome measures by physiotherapists working in outpatient clinics only) [11], for Scotland in 1996 (44%, use of standardised outcome measures in physiotherapy departments) [34], and for the United States in 2009 (48%, standardised outcome measures related to “patients’/ clients’ social, physical, or psychological status” by selected members of the American Physical Therapy Association) [10]. In a survey in 2015 among members of the Austrian physical therapy association, only 17% of participants agreed with the statement “I know of standardised assessment tools in my area” [17]. Although the rate of non-users appears to be relatively low, we assume unsatisfactory usage frequency of MI in the present sample of German physiotherapists in general. This is because 69% of respondents reported using MI in < 80% of their patients. It is well known that physiotherapists may find it difficult to search for and select appropriate MI applicable in their clinical practice [2, 9]. Verheyden and Meyer (2016) [2] listed some methods and channels to support this process, including systematic reviews of MI in specific conditions, information and guidelines provided by educational institutions and physiotherapy associations, and comprehensive books that provide an overview of MI.

The importance of patient prognosis and predicting the likelihood of future outcomes for patients receiving health care interventions [41, 42], and physiotherapy in particular [43, 44], has been strongly advocated in the literature. The results of our survey show that MI were mainly applied for diagnostic purposes and that only 22% of respondents reported the application for prognostic reasons. A possible explanation for this is that high-quality evidence for prognostic factors (in physiotherapy) is limited [45]. Furthermore, the prognostic validity of many MI is unknown or conflicting. This can be illustrated along the Timed Up and Go test (TUG) [46], the most frequently applied measurement of activity limitations in the present survey, which is extensively used by physiotherapists to assess the risk of falling of older people and people with balance deficits over many years. However, recent systematic reviews and meta-analyses have indicated that the TUG has only moderate predictive validity in identifying older people who fall, although the predictive validity varies according to the population and health care setting [47–49]. The lack of prognostic models and the evidence of (sufficient) prognostic validity of many MI has been described for various populations and health-related problems managed by physiotherapists [50–52]. We expect that the assessment and communication of prognostic information in physiotherapy will increase if more evidence on prognostic factors and prognostic validity of MI is available for the physiotherapy profession.

The respondents reported 267 different MI, methods, and devices. The outcomes measured with these MI can be classified by using the framework of the International Classification of Functioning, Disability and Health (ICF) [53]: impairments of body functions and structures, activity limitations, participation restrictions, and contextual factors (personal and environmental). Among the 13 most frequent MI described in one of the German-language textbooks on MI [37–39], only five instruments measure activity limitations restricted to (lower limb) mobility activities (ICF domain d4; TUG, Berg Balance Scale, Performance Oriented Mobility Assessment (POMA), short- distance gait measures, and the 6-min walk test). The DASH/quick-DASH was the only instrument for measuring activity limitations of the upper limb. Among the most frequently mentioned instruments (*n* ≥ 10), no instruments to measure participation, quality of life, or contextual factors were stated, and no instruments were reported to explicitly assess other activity domains relevant to physiotherapy interventions, such as self-care (d5) or domestic life (d6). These results are in accordance with the study by Swinkels et al. (2011) [14], who reported the “visual analogue scale” and the “goniometer” to be the most frequent outcome measures used in private practice.

There are only three patient-reported MI among the most frequently stated ones: (1) 139 participants stated “visual analogue scale”, “numeric analogue scale” or “numeric rating scale”, but it is unclear what construct is measured with these scales, although we assume pain to be the target construct in most statements. (2) Twenty-three participants reported using a “pain scale”, but it is unclear what kind of instrument was used. We assume some form of visual analogue or numeric rating scale to assess pain intensity. (3) The DASH is a patient-reported outcome measure of arm, shoulder and hand disabilities (*n* = 10).

A considerable number of participants provided statements such as “questionnaire” (*n* = 39), “diagnosis sheet” (*n* = 26) or “assessment” (*n* = 11). This indicates a lack of knowledge of the specific name of the instrument or questionnaire (and/or the construct or body structure/function it is intended to measure). For some statements of methods and devices, such as “measuring tape” (*n* = 196) or “weighing scale” (*n* = 18), it is unclear for what purpose/structure/outcome these devices were used. A measuring tape can be used to measure, for example, a limb’s circumference, a timed walking distance, body height, or leg lengths, among others. Statements such as “range of motion” are considered endpoints (outcomes) that can be measured with specific instruments, such as a goniometer or a measuring tape, respectively. This lack of knowledge concerning MI, devices and endpoints was present in the whole data set and might influence the intra- and inter-professional communication, as well as the communication with patients, funders and stakeholders. This issue may be further exacerbated since we observed variability in the denotation of some MI, such as POMA and Tinetti (test, scale, score, etc. [54, 55]), and a bunch of abbreviations that may not be known by intra- and/or inter-professional colleagues (e.g. FABQ for Fear-Avoidance Beliefs Questionnaire, NDI for Neck Disability Index, and KOOS for Knee injury and Osteoarthritis Outcome Score).

The five most frequently applied MI (goniometer, visual analogue scale, manual examination of muscle-strength, TUG, Berg Balance Scale) were usually assessable within a very short time (< 3 Minutes), except for the Berg Balance Scale (10–20 min [56]). We assume that this factor might be important, since in private practice, physiotherapy sessions are scheduled and usually remunerated for 20–30 min. Physiotherapists might not have the time to use MI that require more time, such as questionnaires for patient-reported outcomes of activity and participation. Approximately 30% of the respondents thought that the use of MI was too time-consuming during treatment sessions. This figure is lower than reported in other studies (44% [14] to 75% [10]) and might be explained by the high number of “short” MI used by the respondents in this sample. However, approximately 50% of respondents agreed that the documentation of MI is too time consuming, and that additional financial compensation is required. We assume that addressing these barriers might improve the use of MI in physiotherapy in Germany.

The application of MI was made compulsory by the employers of 29% of respondents. The most frequently reported instruments were comparable to the instruments used by the respondents, with a clear emphasis on performance-based physical measures of body functions and structures.

Duncan and Murray (2012) [9] reviewed the barriers and facilitators to routine outcome measurement for allied health professionals in practice. From 15 papers included, nine used a sample of physiotherapists, and two used a mixed sample of physiotherapists and occupational therapists. The quality of the papers included in this review was mixed. Facilitators and barriers to a routine use of MI were found to exist at individual, managerial and organisational levels.

At the individual level, a positive attitude towards MI and subjective sufficient knowledge and skills to apply MI were strongly pronounced facilitators in the present sample of German physiotherapists. However, approximately every second participants wanted to learn more about the usage of MI. Thus, insufficient knowledge might also be a relevant barrier for some respondents. At a managerial level, respondents generally agreed that individual patient goals can be well integrated into MI. Respondents also agreed that MI can improve a patient’s motivation. At an organisational level, some barriers were pronounced, such as lack of support by the head of the department, time restrictions, and lack of a financial compensation for the use of MI. In conclusion, the limited use of MI reported in the present sample may be due to organisational issues, together with a lack of knowledge and skills needed to apply MI, rather than reasons located at the individual or managerial level.

To ensure successful routine use of MI application in practice, multi-level determinants seem to be important [9, 11, 15, 57]. In agreement with Duncan and Murray (2012) [9], we believe that action is required by organisations, teams and individuals to achieve routine MI application in clinical practice. Organisations should support clinicians by providing appropriate training, sufficient administrative support, and adequate allocation of resources.

Regression analysis showed that an university-based professional degree was positively related to the likelihood of frequently using MI. This result is in agreement with the results reported by others [9, 11, 18]. For example, Copeland et al. [11] reported that physiotherapists with a Master’s degree are twice as likely to use MI as therapists with a lower education. Participants working predominantly in an inpatient setting were approximately four times more likely to use MI than in the outpatient setting. This result might be explained by the higher amount of time physiotherapist usually have in inpatient than in outpatient settings, and that in many inpatient settings, the use of MI is dictated by the employer. Post-hoc analyses show that much more participants working in an inpatient setting reported that MI were dictated by their employers than participants working in outpatient clinics (53% versus 22%).

A further objective of this study was to investigate the facilitators and barriers to the use of an electronic patient health record system. Since 78% of respondents could imagine using an electronic device for a user-friendly patient health record system in clinical practice, we assumed that the respondents had a positive attitude towards such systems. However, most respondents reported at least one barrier or stated problems that might emerge from the clinical usage of electronic devices (such as smartphones or tablets) to document MI. These findings are similar to the opinion of Dutch physiotherapists [58]. The respondents showed a positive attitude towards the implementation of electronic patient records and a strong intention to improve the communication among colleagues while sharing information. Furthermore, they saw the implementation of electronic patient records as a chance to provide a high quality of care. The barriers focused on similar factors to the implementation in the physiotherapy working process and the fact that electronic reporting was more time-consuming.

Most of the reported barriers dealt with organisational issues. These criteria were found in many other reports of electronic medical record implementations all over the world [59, 60]. As it can be seen in the Netherlands, the implementation of electronic patient records is possible, despite many barriers. The support of structural and financial changes from professional bodies and the relief of administrative burden have resulted in visible changes [60].

### Limitations

This is the most recent study to examine the usage of MI by physiotherapists in Germany. The survey was accessible to all physiotherapists working in Germany, and we tried to distribute the survey as far as possible by means of the snowball principle, involving national societies, clinical journals, and other adequate distributers. However, the number of participants (*n* = 522; 0.27%) was relatively low compared to the total number of physiotherapists working in Germany (*n* = 192,000) [26]. This is a crude approximation, since we are not able to calculate an exact response rate. However, we assume that most physiotherapists in Germany were not informed about the survey, although we put much effort into a broad distribution. The use of the Total Design Method as offered by D.A. Dillman [61] might have increased the participation rate, at least from physiotherapists in the cooperation network of our university.

The survey was conducted between November 2014 and February 2015. Although this is the most recent survey on the usage of MI by physiotherapists working in Germany, the usage of MI might have changed in the meantime.

A further limitation is that the survey was only accessible online. This might have increased the participation rate of (younger) physiotherapists and people who were proficient in digital media and online content. However, this method might have deterred therapists working in institutions without internet access or (older) therapists who were not proficient in online content. The mean age of respondents (38 years) was lower than the mean age of the total working population in Germany (43 years) [62]. There is no representative data available for the age distribution of physiotherapists working in Germany.

The percentage of women in the present sample (63%) was lower than in the entire sample of physiotherapists working in Germany (75%) [63] and the distribution of participants throughout the regional states of Germany was not homogeneous. However, there were only two regional states with extreme over- (Hamburg) or under- (Brandenburg) representation. Another source of sampling bias might have been the overrepresentation of therapists with a bachelor degree (22%) or higher (9%) in the present sample compared to representative figures (approximately 3% of German physiotherapists are educated at an academic level [26, 27]). There is evidence, supported by our findings, that physiotherapists with a higher education level tend to use MI more frequently [11, 18]. We further assume that physiotherapists who use MI were more likely to participate in a survey on MI than physiotherapists who do not use MI. Consequently, it is conceivable that the use of MI in the general population of physiotherapists working in Germany is less pronounced than in the present sample.

This study’s data reflects what has been reported and not what has been observed. Thus, recall bias or responding with respect to social desirability might have led to over- or underreporting. We provided a definition of “assessment” in the first section of the survey. However, some participants had divergent associations for this term, since we observed mingling of devices (e.g. measuring tape) and endpoints (e.g. range of motion) together with statements of rather unclear terms, such as “questionnaire” or “assessment”. This indicates lack of knowledge of a sound theoretical background of MI and psychometry in some participants, which should be considered in future surveys. The provision of definitions of the terms mentioned above, together with practical examples, might have increased the validity of the responses.

The questionnaire was built up and based on questions, items and statements used in research on MI application in international research studies. Moreover, the questionnaire has been used in a prior study and was further revised and extended by the authors. Thus, we consider acceptable content (face) validity, but no psychometric evaluation of the survey questionnaire has been performed. It would be desirable to have a psychometrically sound questionnaire on the use of MI, which also has cross-cultural validity, and which can be used to compare the use of MI by physiotherapists, or other health care professionals, between different countries and/or within one country/region over time. The data set of this study may contribute to the development of such a questionnaire.

We used an open-ended question to ask for facilitators and barriers to use an electronic patient health record system in clinical care. The advantage of this method was that respondents were not influenced by predefined categories and answers and we were able to identify a large set of potential facilitators and barriers. However, we had some issues in data interpretation, since some respondents provided statements that were unable to be allocated to one category. For example, the statement “time” without any further explanation might indicate a barrier (“it takes too much time to use an electronic patient health record system”) or a facilitator (“an electronic patient health record system saves time during the process of patient data entry and analysis”).

## Conclusions

MI are used infrequently by physiotherapists working in Germany. Most responders did not use MI for every patient, and the purpose of instrument use was mainly focussed on initial and final assessment of a treatment series. More psychometric evidence on most instruments is needed to inform the prognostic decisions of physiotherapists.

In general, physiotherapists reported a perceived value in MI use and sufficient knowledge for applying appropriate measures. The results of this study, however, indicate insufficient knowledge of the concepts, aims, definitions and terminology of MI. Moreover, most instruments used in clinical care were related to body functions and body structures. Education approaches, at undergraduate and post-graduate levels, should focus on the introduction of patient-centred outcomes, which are located in the domains of activities and participation. An academic degree seems to have a positive influence on the use of MI in practice, since participants with a Bachelor’s degree or higher were four times more likely to use MI more frequently than participants who graduated from a vocational school.

To support the use and application of MI, sufficient time resources and adequate financial compensation are needed. The availability, quality and feasibility of MI need to be improved to facilitate clinical application. The respondents’ attitudes towards electronic patient health record systems in clinical care were rather positive, but the majority of physiotherapists reported reasonable barriers. The findings of this study may help to design and implement electronic patient health record systems in order to support the use, documentation and communication of MI in physiotherapy.

## Additional files

Additional file 1: German-language online survey questionnaire. (PDF 674 kb)

Additional file 2: Description of ways and methods the online survey was published. (PDF 144 kb)

Additional file 3: Distribution of the respondents across the 16 German federal states. (PDF 288 kb)

Additional file 4: The most frequently reported (*n* ≥ 10) measurement instruments, methods and devices. (PDF 120 kb)

Additional file 5: The most frequently reported (*n* ≥ 7) measurement instruments, methods and devices obligated by the employer. (PDF 121 kb)

Additional file 6: Facilitators and barriers to the use of measurement instruments (total and relative frequencies of responses). (PDF 108 kb)

Additional file 7: Facilitators and barriers to the implementation of a user-friendly electronic health record system in one of the following superordinate points according to Wensing et al. (2005), *n* = 309. (PDF 397 kb)

Additional file 8: Summary of all facilitators and barriers to the implementation of a user-friendly electronic health record system according to the classification of Wensing et al. (2005). (PDF 218 kb)

## Abbreviations

AAP: Activities and participation
BFS: Body functions and structures
CHERRIES: Checklist for Reporting Results of Internet E-Surveys
DASH: Disabilities of the Arm, Shoulder and Hand Score
FABQ: Fear-Avoidance Beliefs Questionnaire
ICF: International Classification of Functioning, Disability and Health
KOOS: Knee injury and Osteoarthritis Outcome Score
MI: measurement instrument
NDI: Neck Disability Index
POMA: Performance Oriented Mobility Assessment
QR: Quick Response
STROBE: Strengthening the Reporting of Observational Studies in Epidemiology
TUG: Timed Up and Go test
