# Supplementary material for: Current use of measurement instruments by physiotherapists working in Germany: a cross-sectional online survey
Source: BMC Health Serv Res. 2018 Oct 23;18:810. doi: 10.1186/s12913-018-3563-2 (PMC6199696; doi:10.1186/s12913-018-3563-2)
Supplement: Supplementary file 1 — German-language online survey questionnaire. (PDF 674 kb) [file 12913_2018_3563_MOESM1_ESM.pdf]

Vorschau - nicht drucken

Studienbereich Physiotherapie 13.11.2014 - 11.01.2015
Projektgruppe Assessment-Umfrage

Markieren Sie so: ☐ ☒ ☐ ☐ ☐ Bitte verwenden Sie einen Kugelschreiber oder nicht zu starken Filzstift. Dieser Fragebogen wird maschinell erfasst.
Korrektur: ☐ ■ ☐ ☒ ☐ Bitte beachten Sie im Interesse einer optimalen Datenerfassung die links gegebenen Hinweise beim Ausfüllen.

Regionale Zuordnung

In welchem Land arbeiten Sie?

☐ Deutschland ☐ Schweiz ☐ Österreich
☐ In einem anderen Land

In welchem Bundesland arbeiten Sie?

☐ Baden-Würtemberg ☐ Bayern ☐ Berlin
☐ Brandenburg ☐ Bremen ☐ Hamburg
☐ Hessen ☐ Mecklenburg-Vorpommern ☐ Niedersachsen
☐ Nordrhein-Westfalen ☐ Rheinland-Pfalz ☐ Saarland
☐ Sachsen ☐ Sachsen-Anhalt ☐ Schleswig-Holstein
☐ Thüringen

1 Personenbezogene und arbeitsbezogene Faktoren

1.1 Wie alt sind Sie? (Angabe in Jahren)

1.2 Geschlecht: ☐ weiblich ☐ männlich

1.3 Welchen Ausbildungsgrad besitzen Sie?

1.3.1 Bitte nennen Sie Ihren Ausbildungsgrad.

Bitte wählen Sie den zutreffenden Punkt aus. (Mehrfachnennung möglich; Ergänzung in den Textfeldern möglich)

☐ a) Ausbildung an einer Berufsfachschule ☐ b) Bachelor-Abschluss/ Diplom ☐ c) Master-Abschluss
☐ d) Höhere akademische Bildung

Ergänzung zu a)

Ergänzung zu b)

Ergänzung zu c)

Ergänzung zu d)

1.3.2 Welche abrechnungsfähigen Fortbildungen haben Sie bereits absolviert?

Bitte wählen Sie die zutreffenden Punkte aus. (Mehrfachnennung möglich!)

☐ Keine ☐ Krankengymnastik am Gerät ☐ Manuelle Therapie
☐ Lymphdrainage ☐ Vojta ☐ Propriozeptive neuromuskuläre Fazilitation (PNF)
☐ Bobath ☐ Sonstiges

Erläuterung zu Sonstiges:

Vorschau - nicht drucken

1 Personenbezogene und arbeitsbezogene Faktoren [Fortsetzung]

1.4 Derzeitiges Arbeitsumfeld.

Bitte wählen Sie alle zutreffenden Antworten aus.

☐ Krankenhaus ☐ Rehabilitationseinrichtung ☐ Freie Praxis
☐ Sonstiges

Erläuterung zu Sonstiges:

1.5 Wie viele Patientinnen und Patienten behandeln Sie im Durchschnitt pro Tag?

Bitte wählen Sie eine der folgenden Antworten.

☐ keine Patienten ☐ 1-5 ☐ 6-10
☐ 11-15 ☐ 16-20 ☐ 21-25
☐ >25

1.6 Patientenklientel

1.6.1 Welches Patientenklientel finden Sie hauptsächlich in Ihrem persönlichen Tätigkeitsbereich vor?

(Hinweis: Gewichtung bitte in %. Die Summe der Angaben sollte 100% sein)

	0%	10%	20%	30%	40%	50%	60%	70%	80%	90%	100%
a) Muskuloskelettaler Bereich	☐	☐	☐	☐	☐	☐	☐	☐	☐	☐	☐
b) Neurologischer Bereich	☐	☐	☐	☐	☐	☐	☐	☐	☐	☐	☐
c) Innere Medizin	☐	☐	☐	☐	☐	☐	☐	☐	☐	☐	☐
d) Gemischte Beeinträchtigungen	☐	☐	☐	☐	☐	☐	☐	☐	☐	☐	☐

1.6.2 Bitte Wählen Sie die Altersgruppe Ihres Patientenklientels. (Mehrfachnennung möglich!)

☐ Kleinkinder (< 6 Jahre) ☐ Kinder (6 - 13 Jahre) ☐ Jugendliche (14 -17 Jahre)
☐ Erwachsene (18 - 65 Jahre) ☐ höheres Lebensalter (> 65 Jahre)

1.7 Wie viele Stunden in der Woche arbeiten Sie im Durchschnitt als Physiotherapeutin/Physiotherapeut?

(Bitte auf volle Stundenangaben runden. Bsp.: 38 Stunden/Woche)

1.8 Wie viele Jahre Berufserfahrung haben Sie als Physiotherapeutin/ Physiotherapeut?

☐ < 1 Jahr ☐ 1-3 Jahre ☐ 4-10 Jahre
☐ 11-15 Jahre ☐ > 15 Jahre

2 Verwendung von Messinstrumenten

Begriffsdefinitionen:

Assessments: Verfahren, um Merkmale und Eigenschaften auf systematische Weise zu erfassen und auf einer definierten Skala festzuhalten. Assessment wird dabei dem Begriff Messinstrument gleichgesetzt, dazu zählen auch Fragebögen. Standardisierte objektive Assessments dienen der Diagnostik/Befunderhebung, Ergebnismessung und Prognosestellung (aus Wirz et al. 2014).

Interdisziplinär: "mehrere Disziplinen umfassend; die Zusammenarbeit mehrerer Disziplinen betreffend" (Duden).

2.1 Ich verwende generell Messinstrumente.

☐ Ja, ich verwende Messinstrumente ☐ Nein, ich verwende keine Messinstrumente

Vorschau - nicht drucken

2 Verwendung von Messinstrumenten [Fortsetzung]

2.2 Ich verwende Messinstrumente vor allem... (Bitte wählen Sie alle zutreffenden Antworten aus.)

- ☐ für diagnostische Zwecke
- ☐ für prognostische Zwecke
- ☐ als Test in der ersten Therapieeinheit
- ☐ als Zwischenbefund
- ☐ als Test in der letzten Therapieeinheit
- ☐ Weiteres

Erläuterung zu Weiteres:

2.3 Wie viele verschiedene Messinstrumente verwenden Sie?

(Bitte wählen Sie eine der folgenden Antworten aus.)

- ☐ 1-2 verschiedene Messinstrumente
- ☐ 3-5 verschiedene Messinstrumente
- ☐ 6-10 verschiedene Messinstrumente
- ☐ 11-20 verschiedene Messinstrumente
- ☐ >20 verschiedene Messinstrumente

2.4 Ich benutze Messinstrumente bei ca. ...% meiner Patienten.

(Tragen Sie bitte auf der Skala von 0-100% ein: 0 %: bei keinem Patienten, 100 %: bei allen Patienten.)

0%	10%	20%	30%	40%	50%	60%	70%	80%	90%	100%
☐	☐	☐	☐	☐	☐	☐	☐	☐	☐	☐

Vorschau - nicht drucken

Vorschau - nicht drucken

2 Verwendung von Messinstrumenten [Fortsetzung]

2.5 Von meinem Arbeitgeber ist die Benutzung von Messinstrumenten vorgegeben.

☐ Nein ☐ Ja, und zwar:

Bitte geben Sie Ihre Antwort(en) hier ein:
Bitte die Namen der Messinstrumente ausschreiben.

Messinstrument 1:

Messinstrument 2:

Messinstrument 3:

Messinstrument 4:

Messinstrument 5:

Messinstrument 6:

2.6 Die Messinstrumente, die ich am häufigsten nutze, sind...

(Angefangen mit dem Messinstrument, das am meisten genutzt wird. Bitte die Namen der Messinstrumente ausschreiben)

Bitte geben Sie Ihre Antwort(en) hier ein:

Messinstrument 1:

Messinstrument 2:

Messinstrument 3:

Messinstrument 4:

Messinstrument 5:

Messinstrument 6:

Vorschau - nicht drucken

Vorschau - nicht drucken

3 Förderfaktoren und Barrieren

Bitte bewerten Sie die nachfolgenden Aussagen mit der angeführten Skala.

3.1 Therapeutenperspektive

	Trifft nicht zu	Trifft eher nicht zu	neutral	Trifft eher zu	Trifft zu	Kann ich nicht beurteilen
Ich bin vom klinischen Nutzen der Messinstrumente überzeugt.	☐	☐	☐	☐	☐	☐
Mir fehlt die tägliche Routine in der Anwendung von Messinstrumenten.	☐	☐	☐	☐	☐	☐
Ich bin davon überzeugt, dass der Einsatz von Messinstrumenten die Qualität meiner Behandlung verbessert.	☐	☐	☐	☐	☐	☐

3.2 Wissen und Fertigkeiten

Bitte wählen Sie die zutreffende Antwort für jeden Aussage aus.

	Trifft nicht zu	Trifft eher nicht zu	neutral	Trifft eher zu	Trifft zu	Kann ich nicht beurteilen
Ich verfüge über ausreichend Wissen, um Messinstrumente zu benutzen.	☐	☐	☐	☐	☐	☐
Ich bin im Umgang mit Messinstrumenten gut ausgebildet.	☐	☐	☐	☐	☐	☐
Es gibt so viele verschiedene Messinstrumente, ich weiß nicht, welche ich davon benutzen soll.	☐	☐	☐	☐	☐	☐
Es ist für mich wichtig, mehr über den Gebrauch von Messinstrumenten zu erfahren.	☐	☐	☐	☐	☐	☐

3.3 Therapeutisches Setting

Bitte wählen Sie die zutreffende Antwort für jeden Punkt aus.

	Trifft nicht zu	Trifft eher nicht zu	neutral	Trifft eher zu	Trifft zu	Kann ich nicht beurteilen
In der Verwendung von Messinstrumenten können die individuellen Behandlungsziele der Patienten gut integriert werden.	☐	☐	☐	☐	☐	☐
Patienten werden durch die Verwendung von Messinstrumenten motiviert.	☐	☐	☐	☐	☐	☐

Vorschau - nicht drucken

Vorschau - nicht drucken

3 Förderfaktoren und Barrieren [Fortsetzung]

Die Verwendung von Messinstrumenten verbessert die Kommunikation zwischen Therapeut/in und Patient/in.	☐	☐	☐	☐	☐	☐
Patienten finden die Verwendung von Messinstrumenten zu zeitaufwendig.	☐	☐	☐	☐	☐	☐
Das Patientenklientel, das ich behandle, ist für den Einsatz von Messinstrumenten ungeeignet.	☐	☐	☐	☐	☐	☐

3.4 Organisationsstrukturen

Bitte wählen Sie die zutreffende Antwort für jeden Punkt aus.

	Trifft nicht zu	Trifft eher nicht zu	neutral	Trifft eher zu	Trifft zu	Kann ich nicht beurteilen
Die Therapieleitung meiner Einrichtung unterstützt den Gebrauch von Messinstrumenten.	☐	☐	☐	☐	☐	☐
Die Ergebnisse der Messinstrumente bieten eine stärkere Argumentationsgrundlage gegenüber Kostenträgern.	☐	☐	☐	☐	☐	☐
Die Verwendung von Messinstrumenten während der Behandlung ist zu zeitaufwendig.	☐	☐	☐	☐	☐	☐
Die Dokumentation der Messergebnisse ist sehr aufwendig.	☐	☐	☐	☐	☐	☐
Der Einsatz von Messinstrumenten erfordert eine zusätzliche finanzielle Entschädigung.	☐	☐	☐	☐	☐	☐

3.5 Clinical Reasoning

Bitte wählen Sie die zutreffende Antwort für jeden Punkt aus.

	Trifft nicht zu	Trifft eher nicht zu	neutral	Trifft eher zu	Trifft zu	Kann ich nicht beurteilen
Die Verwendung von Messinstrumenten hat einen positiven Einfluss auf meinen klinischen Denkprozess/ Clinical-Reasoning (inkl. der Überprüfung von Hypothesen).	☐	☐	☐	☐	☐	☐
Die Messinstrumente unterstützen die Spezifizierung meiner physiotherapeutischen Diagnose.	☐	☐	☐	☐	☐	☐

Vorschau - nicht drucken

Vorschau - nicht drucken

3 Förderfaktoren und Barrieren [Fortsetzung]

Mit der Verwendung von Messinstrumenten fällt es mir leichter, einen konkreten und individuellen Behandlungsplan für meine Patienten zu erstellen.	☐	☐	☐	☐	☐	☐
Messinstrumente helfen mir dabei, meine Behandlung kontinuierlich an den sich wechselnden Status der Patienten anzupassen.	☐	☐	☐	☐	☐	☐
Ich benutzte Messinstrumente als Re-Test, um die Wirksamkeit meiner Behandlungsstrategie zu überprüfen.	☐	☐	☐	☐	☐	☐
Es ist sehr schwer, die Ergebnisse der Messinstrumente zu interpretieren.	☐	☐	☐	☐	☐	☐

Vorschau - nicht drucken

Vorschau - nicht drucken

3 Förderfaktoren und Barrieren [Fortsetzung]

3.6 Interdisziplinäres Handeln

Bitte wählen Sie die zutreffende Antwort für jeden Punkt aus.

	Trifft nicht zu	Trifft eher nicht zu	neutral	Trifft eher zu	Trifft zu	Kann ich nicht beurteilen
Die Verwendung von Messinstrumenten verbessert die interdisziplinäre Kommunikation.	☐	☐	☐	☐	☐	☐
Die Ergebnisse der physiotherapeutischen Messinstrumente haben einen entscheidenden Einfluss auf die interdisziplinäre Umsetzung der individuellen Patientenziele.	☐	☐	☐	☐	☐	☐

3.7 Gibt es für Sie zusätzlich zu den oben genannten Aussagen positive Faktoren, die Ihnen bei der Verwendung von Messinstrumenten wichtig sind?

Bitte geben Sie Ihre Antwort hier ein:

3.8 Gibt es für Sie zusätzlich zu den oben genannten Aussagen weitere Probleme, die Ihnen bei der Verwendung von Messinstrumenten begegnen?

Bitte geben Sie Ihre Antwort hier ein:

3.9 Technische Unterstützung

Momentan wird die elektronische/digitale Dokumentation von Assessments diskutiert, und auch teilweise schon angewendet.

Vorschau - nicht drucken

Vorschau - nicht drucken

3 Förderfaktoren und Barrieren [Fortsetzung]

3.9.1 Können Sie sich grundsätzlich vorstellen, eine **anwenderfreundliche**, computergestützte Dokumentation (z.B. Smartphone, Tablet, Mini-Computer) im praktischen Alltag zu verwenden?

☐ Ja ☐ Nein

3.9.2 Welche Förderfaktoren und Barrieren sehen sie bei der elektronischen/digitalen Dokumentation.

Bitte geben Sie Ihre Antwort hier ein:

Vorschau - nicht drucken

Vorschau - nicht drucken

4 Möglicher Schulungsbedarf

4.1 Welche klinischen Merkmale und Eigenschaften sind Ihnen bei der Behandlung Ihrer Patienten wichtig? Nennen Sie bitte konkrete Beispiele.

Bitte geben Sie Ihre Antwort hier ein:

4.2 Welche methodischen Inhalte wären für Sie in einer Schulung interessant?

Bitte geben Sie Ihre Antwort hier ein:

4.3 Welche Empfehlungen/ Wünsche möchten Sie uns mit auf den Weg geben, um ein passendes Schulungsprogramm für Sie anzubieten?

Bitte geben Sie Ihre Antwort hier ein:

Herzlichen Dank für Ihre Teilnahme!

Vorschau - nicht drucken

4 Möglicher Schulungsbedarf [Fortsetzung]

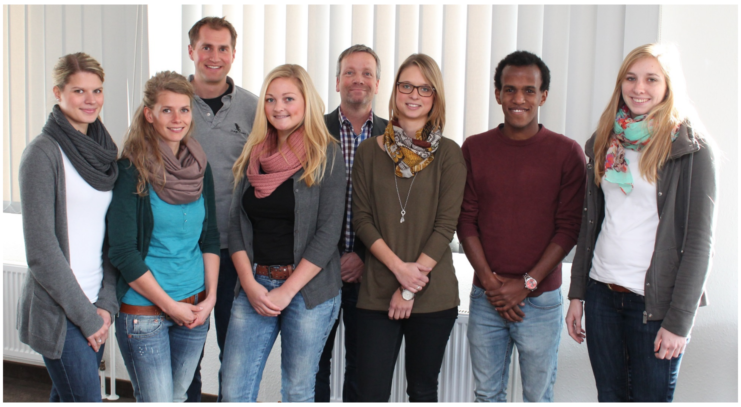

Vorschau - nicht drucken
