# Supplementary material for: Current use of measurement instruments by physiotherapists working in Germany: a cross-sectional online survey
Source: BMC Health Serv Res. 2018 Oct 23;18:810. doi: 10.1186/s12913-018-3563-2 (PMC6199696; doi:10.1186/s12913-018-3563-2)
Supplement: Supplementary file 2 — Description of ways and methods the online survey was published. (PDF 144 kb) [file 12913_2018_3563_MOESM2_ESM.pdf]

Additional file 2: Description of ways and methods the online survey was published

Institution/journal/society	Way of launching/media/publication/distribution					
	Homepage post/ advertisement	Press release	Journal announcement	Facebook post	Newsletter (for subscribers)	Flyer display on the booth at an exhibition
Hochschule für Gesundheit (University of Applied Sciences; host institution)	X	X				
Medica ("world forum for medicine") trade fair, Düsseldorf, November 2014						X
Zeitschrift für Physiotherapeuten (at this time the official Journal of the German physiotherapy association)			X	X		
Physiopraxis (German journal for physiotherapy practice)			X	X	X	
Physio-Journal (German journal for physiotherapy practice)				X		
Physio-Deutschland (German Association for Physical Therapy)	X			X		
Physio Akademie (training institute of the German Association for Physical Therapy)	X			X	X	
IFK e.V. (German association for self-employed physical therapists)	X			X		
VeBID (Society of the Bobath instructors in Germany and Austria)					X	
AG Manuelle Therapie (German society for manual therapy)				X		
FOMT (training institute for orthopaedic medicine and manual therapy)	X			X	X	
Opta data (private sector)					X	

**Institution/journal/society**	**Way of launching/ media**
Hochschule für Gesundheit	**A, B**
Medica (“world forum for medicine”)	**F**
Zeitschrift für Physiotherapeuten (at this time the official Journal of the German physiotherapy association)	**C, D**
Physiopraxis (German journal for physiotherapy practice)	**C, D, E**
Physio-Journal (German journal for physiotherapy practice)	**D**
Physio-Deutschland (German Association for Physical Therapy)	**A, D**
Physio Akademie (training institute of the German Association for Physical Therapy)	**A, D, E**
IFK e.V. (German association for self-employed physical therapists)	**A, D**
VeBID (Society of the Bobath instructors in Germany and Austria)	**E**
AG Manuelle Therapie (German society for manual therapy)	**D**
FOMT (training institute for orthopaedic medicine and manual therapy)	**A, D, E**
Opta data (private sector …)	**E**
Detl Physioswiss	
twitter	
A = Homepage post/ advertisement; B = Press release; C = Journal announcement; D = Facebook post; E = Newsletter (for subscribers); F = Flyer display on the booth at an exhibition; G = twitter	
