# Supplementary material for: Current use of measurement instruments by physiotherapists working in Germany: a cross-sectional online survey
Source: BMC Health Serv Res. 2018 Oct 23;18:810. doi: 10.1186/s12913-018-3563-2 (PMC6199696; doi:10.1186/s12913-018-3563-2)
Supplement: Supplementary file 3 — Distribution of the respondents across the 16 German federal states. (PDF 288 kb) [file 12913_2018_3563_MOESM3_ESM.pdf]

Additional file 3: Distribution of the respondents across the 16 German federal states

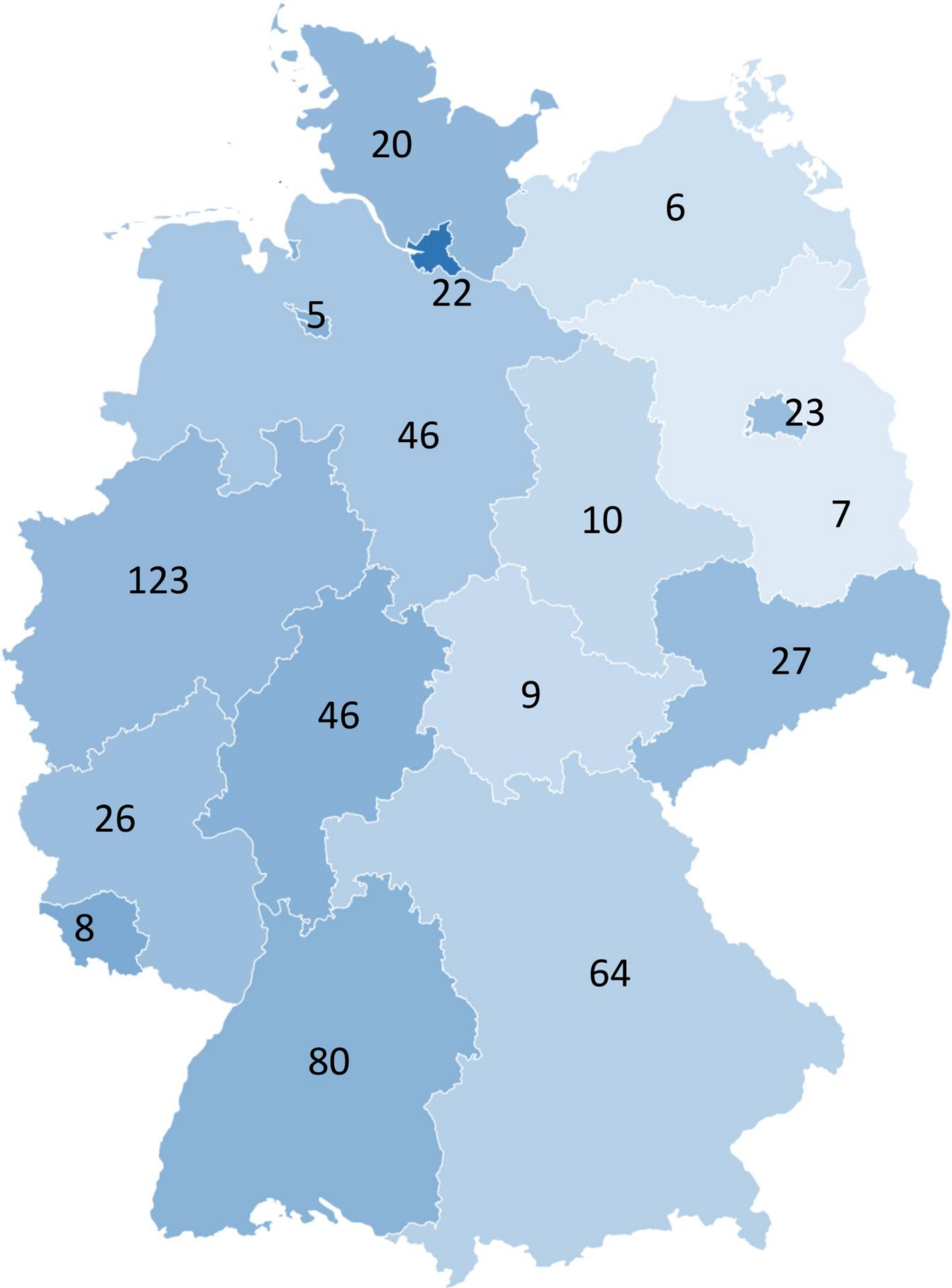

Number of respondents (n = 522) according to regional states (n = 16) across Germany. Colors indicate the rate between the number of responding physiotherapist and the number of inhabitants in each regional state, with dark and bright blue shades indicating higher and lower rates, respectively. For example, Hamburg has the highest rate (0.00123%) with 22 respondents and 1.787 million inhabitants, and Brandenburg has the lowest rate (0,00028%) with 7 respondents and 2.485 million inhabitants (Data source: Statistisches Bundesamt; www.destatis.de; "Bundesländer mit Hauptstädten nach Fläche, Bevölkerung und Bevölkerungsdichte am 31.12.2016").
