# Supplementary material for: Current use of measurement instruments by physiotherapists working in Germany: a cross-sectional online survey
Source: BMC Health Serv Res. 2018 Oct 23;18:810. doi: 10.1186/s12913-018-3563-2 (PMC6199696; doi:10.1186/s12913-018-3563-2)
Supplement: Supplementary file 4 — The most frequently reported (n ≥ 10) measurement instruments, methods and devices. (PDF 120 kb) [file 12913_2018_3563_MOESM4_ESM.pdf]

Additional file 4: The most frequently reported (n ≥ 10) measurement instruments

**The most frequently reported (n ≥ 10) measurement instruments**						
Ranking	Measurement instrument, device or method	n	Percent (95% CI)	Measurement instrument*	ICF domain	
					BFS	AAP
1.	Goniometer	254	49% (44 – 53)	yes	X	
2.	Measuring tape/ruler	196	38% (33 – 42)	no		
3.	Visual analogue scale/numeric rating scale/numeric analogue scale	139	27% (22 – 31)	yes	X	
4.	Manual examination of muscle-strength	54	10% (6 – 15)	yes	X	
5.	Timed Up and Go test	53	10% (6 – 14)	yes		X
6.	Range of motion/neutral zero method	42	8% (4 – 12)	no		
7.	Questionnaire	39	7% (3 – 12)	no		
8.	Reflex hammer	35	7% (2 – 11)	no		
9.	Diagnosis sheet/patient report chart/findings sheet	26	5% (1 – 9)	no		
10.	Pain scale	23	4% (0 – 9)	no		
11.	Berg Balance Scale	23	4% (0 – 9)	yes		X
12.	Measure of circumference	21	4% (0 – 8)	yes	X	
13.	Weighing scale	18	3% (0 – 8)	no		
14.	Dynamometer/devices to measure muscle strength (hand held)	16	3% (0 – 7)	yes	X	
15.	Finger-floor distance	16	3% (0 – 7)	yes	X	X
16.	Performance Oriented Mobility Assessment	16	3% (0 – 7)	yes		X
17.	Blood-pressure measuring device	15	3% (0 – 7)	yes	X	
18.	Gait measures (short distance <10m or <10sec)	14	3% (0 – 7)	yes		X
19.	6 minute walk test	12	2% (0 – 7)	yes		X
20.	Assessments	11	2% (0 – 6)	no		
21.	DASH/Quick-DASH	10	2% (0 – 6)	yes	X	X

Abbreviations: n = number of reports; CI = confidence interval; ICF = International Classification of Functioning, Disability and Health; BFS = body functions and structures; AAP = activities and participation; DASH = Disabilities of the Arm, Shoulder and Hand.

* as described in one of the German-language textbooks on measurement instruments published by Schädler et al. [37], Oesch et al. [38] and Büsching et al. [39].
