# Supplementary material for: Current use of measurement instruments by physiotherapists working in Germany: a cross-sectional online survey
Source: BMC Health Serv Res. 2018 Oct 23;18:810. doi: 10.1186/s12913-018-3563-2 (PMC6199696; doi:10.1186/s12913-018-3563-2)
Supplement: Supplementary file 5 — The most frequently reported (n ≥ 7) measurement instruments, methods and devices obligated by the employer. (PDF 121 kb) [file 12913_2018_3563_MOESM5_ESM.pdf]

Additional file 5: The most frequently reported (n ≥ 7) measurement instruments obligated by the employer

**The most frequently reported (n ≥ 7) measurement instruments obligated by the employer**						
Ranking	Measurement instrument, device or method	n	Percent (95% CI)	Measurement instrument*	ICF-domain	
					BFS	AAP
1.	Goniometer	79	15% (12 – 18)	yes	X	
2.	Measuring tape/ruler	45	9% (6 – 12)	no		
3.	Visual analogue scale/numeric rating scale/numeric analogue scale	45	9% (6 – 12)	yes	X	
4.	Timed Up and Go test	31	6% (3 – 9)	yes		X
5.	Manual examination of muscle-strength	16	3% (0 – 6)	yes	X	
6.	Range of motion/neutral zero method	16	3% (0 – 6)	no		
7.	Diagnosis sheet/patient report chart/findings sheet	15	3% (0 – 6)	no		
8.	Measure of circumference	15	3% (0 – 6)	yes	X	
9.	Questionnaire	13	2% (0 – 6)	no		
10.	Reflex hammer	12	2% (0 – 5)	no		
11.	Berg Balance Scale	11	2% (0 – 5)	yes		X
12.	Dynamometer/devices to measure muscle strength (hand held)	10	2% (0 – 5)	yes	X	
13.	Assessments	9	2% (0 – 5)	no		
14.	Gait measures (short distance <10m or <10sec)	9	2% (0 – 5)	yes		X
15.	Performance Oriented Mobility Assessment	9	2% (0 – 5)	yes		X
16.	Pain scale	9	2% (0 – 5)	no		
17.	6 minute walk test	8	2% (0 – 5)	yes		X
18.	Computer-supported measure of muscle-strength	8	2% (0 – 5)	no		
19.	Anamnesis/anamnesis sheet	7	1% (0 – 4)	no		
20.	Barthel Index	7	1% (0 – 4)	yes		X
21	Blood-pressure measuring device	7	1% (0 – 4)	yes	X	

Abbreviations: n = number of reports; CI = confidence interval; ICF = International Classification of Functioning, Disability and Health; BFS = body functions and structures; AAP = activities and participation
* as described in one of the German-language textbooks on measurement instruments published by Schädler et al. [37], Oesch et al. [38] and Büsching et al. [39].
