# Supplementary material for: Current use of measurement instruments by physiotherapists working in Germany: a cross-sectional online survey
Source: BMC Health Serv Res. 2018 Oct 23;18:810. doi: 10.1186/s12913-018-3563-2 (PMC6199696; doi:10.1186/s12913-018-3563-2)
Supplement: Supplementary file 6 — Facilitators and barriers to the use of measurement instruments (total and relative frequencies of responses). (PDF 108 kb) [file 12913_2018_3563_MOESM6_ESM.pdf]

Additional file 6: Facilitators and barriers to the use of measurement instruments (total and relative frequencies of responses)

	Frequency of responses (n)						sum	valid	Frequency of responses (percent)					sum
	agree	rather agree	neutral	rather disagree	disagree	missing	(n)	(n)	agree	rather agree	neutral	rather disagree	disagree	(%)
**Attitudes and beliefs of the therapist**														
I am convinced of the benefits of measurement instruments for clinical care (missing = 15)	272	118	73	33	11	15	522	507	54	23	14	7	2	100
I miss the routine of using measurement instruments in daily clinical practice (missing = 9)	57	60	79	132	185	9	522	513	11	12	15	26	36	100
I am convinced that the use of measurement instruments improves the quality of my treatment (missing = 12)	252	111	52	50	45	12	522	510	49	22	10	10	9	100
**Skills and knowledge**														
I have sufficient knowledge to use measurement instruments (missing = 8)	262	130	82	31	9	8	522	514	51	25	16	6	2	100
I have sufficient skills to apply measurement instruments (missing = 7)	202	151	92	51	19	7	522	515	39	29	18	10	4	100
There are so many different instruments, I do not know which one to use (missing =24)	26	67	120	143	142	24	522	498	5	13	24	29	29	100
**Therapeutic setting**														
It is feasible to integrate a patient's individual treatment-goals in the application of measurement instruments (missing = 22)	200	146	109	35	10	22	522	500	40	29	22	7	2	100
Patients are motivated through the use of measurement instruments (missing = 15)	203	188	66	35	15	15	522	507	40	37	13	7	3	100
Usage of mesurement instruments improves the communication between the therapist and the patient (missing = 12)	176	172	100	40	22	12	522	510	35	34	20	8	4	100
Patients perceive the use of measurement instruments too time consuming (missing = 50)	31	71	128	163	79	50	522	472	7	15	27	35	17	100
The kind of patients I treat are unsuitable for the use of measurement instruments (missing = 20)	18	25	85	167	207	20	522	502	4	5	17	33	41	100
**Organisational structures**														
The head of my institution/department supports the use of measurement instruments (missing = 18)	217	63	137	44	43	18	522	504	43	13	27	9	9	100
Using measurement instruments strengthen negotiations with insurers (missing = 43)	235	121	57	31	35	43	522	479	49	25	12	6	7	100
Using measurement instruments during treatment sessions is too time consuming (missing = 14)	61	110	109	128	100	14	522	508	12	22	21	25	20	100
The documentation of outcomes is too time consuming (missing = 13)	88	168	84	98	71	13	522	509	17	33	17	19	14	100
Using measurement instruments requires additional financial compensation (missing = 37)	164	102	87	64	68	37	522	485	34	21	18	13	14	100
**Clinical reasoning process**														
The use of measurement instruments impacts my clinical reasoning in a positive way, including the testing of hypotheses (missing = 26)	213	165	67	29	22	26	522	496	43	33	14	6	4	100
Measurement instruments support the specification of my physiotherapeutic diagnosis (missing = 19)	195	170	76	39	23	19	522	503	39	34	15	8	5	100
The usage of measurement instruments makes it easier for me to complie a specific and individual treatment plan for my patients (missing = 18)	153	157	96	66	32	18	522	504	30	31	19	13	6	100
Measurement instruments support constant matching of my treatment strategy with the fluctuent health state of my patients (missing = 19)	161	162	100	52	28	19	522	503	32	32	20	10	6	100
I use measurement instruments as a re-test to evaluate the efficacy of my treatment strategy (missing = 15)	263	132	47	35	30	15	522	507	52	26	9	7	6	100
The interptretation of test results is very difficult (missing = 26)	13	39	108	198	138	26	522	496	3	8	22	40	28	100
**Interprofessional approach**														
The usage of measurement instruments improves inter-disciplinary communication (missing = 40)	175	157	100	32	18	40	522	482	36	33	21	7	4	100
The outcomes generted with physiotherapeutic measurement instruments have a substantial impact on the inter-disciplinary achievement of individual patient goals (missing = 49)	118	126	155	47	27	49	522	473	25	27	33	10	6	100
