# Supplementary material for: Current use of measurement instruments by physiotherapists working in Germany: a cross-sectional online survey
Source: BMC Health Serv Res. 2018 Oct 23;18:810. doi: 10.1186/s12913-018-3563-2 (PMC6199696; doi:10.1186/s12913-018-3563-2)

Additional file 7: Facilitators and barriers to the implementation of a user-friendly electronic health record system in one of the following superordinate points according to Wensing et al. (2005), n = 309

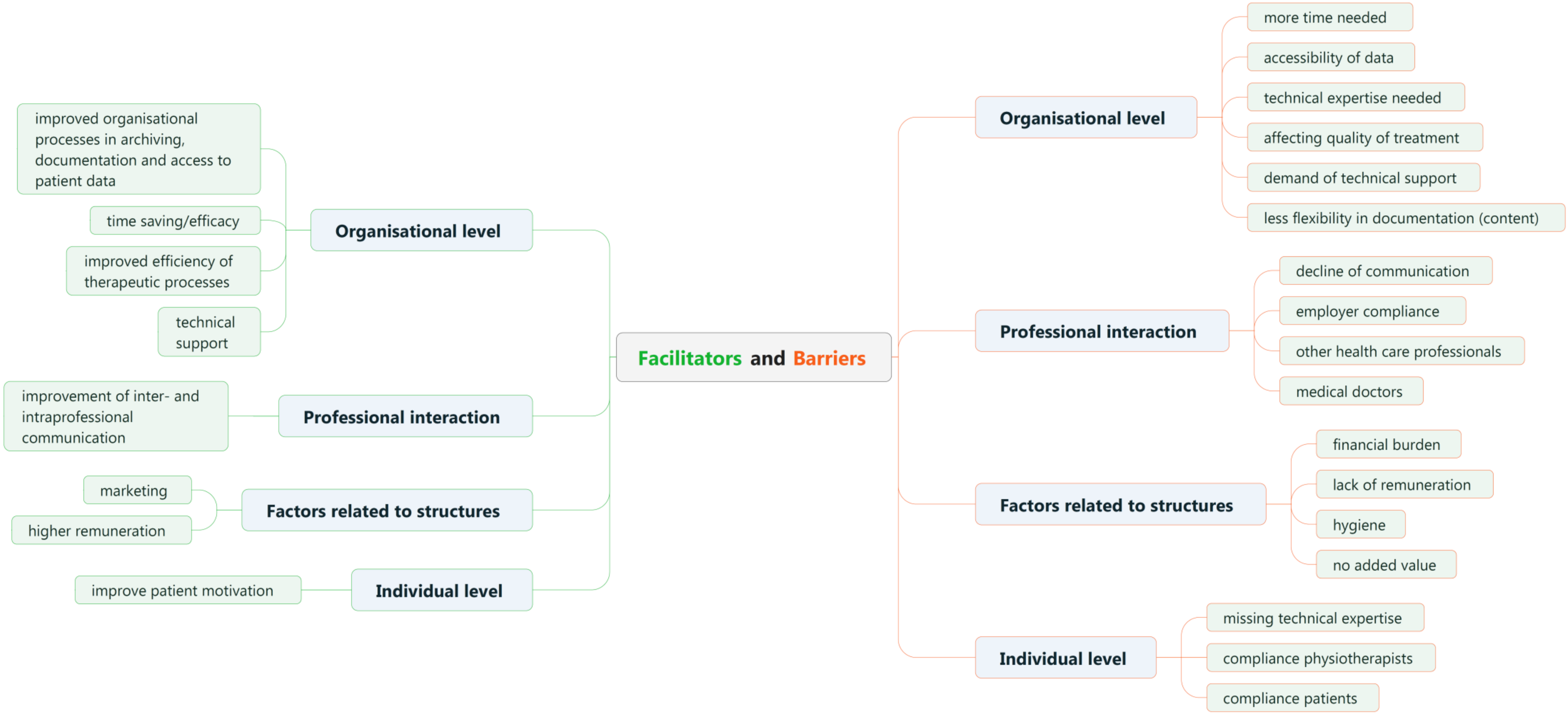

Supplement: Supplementary file 7 — Facilitators and barriers to the implementation of a user-friendly electronic health record system in one of the following superordinate points according to Wensing et al. (2005), n = 309. (PDF 397 kb) [file 12913_2018_3563_MOESM7_ESM.pdf]
