# Supplementary material for: Current use of measurement instruments by physiotherapists working in Germany: a cross-sectional online survey
Source: BMC Health Serv Res. 2018 Oct 23;18:810. doi: 10.1186/s12913-018-3563-2 (PMC6199696; doi:10.1186/s12913-018-3563-2)
Supplement: Supplementary file 8 — Summary of all facilitators and barriers to the implementation of a user-friendly electronic health record system according to the classification of Wensing et al. (2005). (PDF 218 kb) [file 12913_2018_3563_MOESM8_ESM.pdf]

Additional file 8: Summary of all facilitators and barriers to the implementation of a user-friendly electronic health record system according to the classification of Wensing et al. (2005)

Facilitators

Organisational level	**Improvement of organisational processes**	
	Organisational resources	access to patient records
		simplification of archiving and storage
	Organisational processes	analysis and documentation
		readability
		electronic appointment allocation
	Organisational structures	paper-free documentation
	**Time-effective manner**	
	Organisational processes	faster documentation and therapy report
		simplified organization
		faster communication with other health care professions
	Organisational resources	accessibility tools/input assistance and text blocks
	**Improved efficiency of therapeutic processes**	
	Organisational processes	improvement of quality and transparency of therapy
		administration of patient-reported measurements/questionnaires in advance
		demonstrating treatment progress and success
	Organizational resources	make use of assessments
		photo documentation
	Organisational structures	clinical reasoning
		standardisation
	**Technical support**	
	Organisational resources	intuitive handling
		compliance with documentation requirement
Professional interaction	**Improvement of communication**	
	Structure of professional networks	with other professions
		with other physiotherapists
Factors related to structures	**Quality**	
	Societal factors	marketing/professional behaviour
	Regulations	higher remuneration (as motivation)
Individual level	**Communication**	
	Motivational factors	motivating patients

Barriers

	**Time-consuming**	
	Organisational resources	induction and training programmes
		loss of time during treatment
		faster with handwritten documentation
	Organisational structures	transmission of handwritten notes
		data entry
	**Accessibility of data / technical dependency**	
	Organisational resources	internet access
		risk of data loss
		access to hardware
	Organisational structures	synchronisation is necessary
		dependent on technology (risk of system break down)
Organisational level	**Technical expertise**	
	Organisational processes	data protection
		training education programmes
	**Quality of treatment**	
	Organisational structures	loss of individuality
		data manipulation
		malpractice due to typing error
	**Demand of technical support**	
	Organisational resources	storage capacity
		updates needed
	**Flexibility**	
	Organisational structures	lack of individuality of the programs/ missing response options
		marking in body chart
Professional interaction	**Decline of communication**	
	Interaction in professional teams	patient-therapist interaction
	**Employer**	
	Structure of professional networks	compliance and willingness
	**Medical doctors**	
	Interaction in professional teams	understanding/interpretation of results
	Structure of professional networks	written and printed documentation accepted only
	**Other health care professionals**	

Factors related to structures	**Costs**	
	Financial incentives	purchase/acquisition cost
		hardware investments
		maintenance
		Training and education
		profitability/cost effectiveness
		loss or theft
	**Remuneration**	
	Societal factors	lack of remuneration
	**Hygiene**	
	Regulations	hardware
	**No added value**	
	Societal factors	unusable results for daily practice
		lack of recognition
	**Excessive use of digital media**	
	Societal factors	inordinate focus on digital media
Individual level	**Technical expertise**	
	Cognitive factors	higher aged therapists
		initial training needed
	**Compliance physiotherapists**	
	Cognitive factors	older physiotherapists
	Motivational factors	personal attitude to technology
		fear of being controlled
	**Patients**	
	Motivational factors	compliance
	Cognitive factors	comprehensibility
		technical expertise
